# Pro-Inflammatory Mediators and Apoptosis Correlate to rt-PA Response in a Novel Mouse Model of Thromboembolic Stroke

**DOI:** 10.1371/journal.pone.0085849

**Published:** 2014-01-20

**Authors:** Saema Ansar, Eva Chatzikonstantinou, Rushani Thiagarajah, Laurent Tritschler, Marc Fatar, Michael G. Hennerici, Stephen Meairs

**Affiliations:** Department of Neurology, University Medicine Mannheim, Heidelberg University, Heidelberg, Germany; National University of Singapore, Singapore

## Abstract

**Background:**

A recent study suggests that patients with persistent occlusion of the middle cerebral artery (MCA) following treatment with recombinant tissue plasminogen activator (rt-PA) have better outcomes than patients with MCA occlusion not receiving rt-PA. We performed a study to elucidate possible mechanisms of this finding in a new model of thromboembolic stroke closely mimicking human pathophysiology.

**Methods:**

Thromboembolic stroke was induced by local injection of thrombin directly into the right MCA of C57 black/6J mice. Rt-PA was administered 20 and 40 min after clot formation. The efficiency of rt-PA to induce thrombolysis was measured by laser Doppler. After 24 h, all animals were euthanized and interleukin (IL)-6, tumor necrosis factor-alpha (TNF-α), matrix metalloproteinase (MMP)-9, Caspase-3, hsp 32 and hsp 70 protein levels were investigated by immunofluorescence. Presence of hemorrhage was verified and infarct volume was measured using histology.

**Results:**

Thrombin injection resulted in clot formation giving rise to cortical brain infarction. Early rt-PA treatment starting at 20 min after the clot formation resulted in 100% recanalization. However, rt-PA-induced thrombolysis dissolved the clot in only 38% of the animals when administered 40 min after clot formation. Protein levels of IL-6, TNF-α, MMP-9, Caspase-3, hsp 32 and hsp 70 were increased after MCAO, whereas treatment with rt-PA attenuated the expressions of inflammatory markers in those animals where the thrombolysis was successful. In addition, the infarct size was significantly reduced with rt-PA treatment compared to non-treated MCAO, regardless of whether MCA thrombolysis was successful.

**Conclusions:**

The present study demonstrates a clear correlation of the protein expression of inflammatory mediators, apoptosis and stress genes with the recanalization data after rt-PA treatment. In this model rt-PA treatment decreases the infarct size regardless of whether vessel recanalization is successful.

## Introduction

Sudden occlusion of a cerebral blood vessel by a thrombus or embolism initiates a complex process of events that includes excitotoxity, oxidative stress, microvascular injury, blood brain barrier dysfunction and postischemic inflammation that ultimately leads to cell death. Although several mechanisms are involved in the pathophysiology of stroke, increasing evidence shows that inflammation is a key contributor to the pathophysiology of cerebrovascular diseases [1]–[3] and this correlates with the outcome of the patient. Inflammation contributes to breakdown of the blood-brain barrier (BBB) which promotes the formation of brain edema and contributes to acute mortality in stroke [4]. In the acute phase of stroke activation of cytokines [interleukin-1 beta (IL-1ß), interleukin 6 (IL-6) and tumor necrosis factor-alpha (TNF-α)] [5], chemokines [monocyte chemostatic protein-1 (MCP-1) and macrophage inflammatory protein-1 alpha], [6] matrix metalloproteinases (MMPs) [7], adhesion molecules ICAM-1, P-selectin, E-electin and toxic molecules such as nitric oxide, free radicals, apoptosis (Caspase 3), and stress genes (hsp 70 and hsp 32) occurs.

The only approved therapy for acute thromboembolic stroke remains thrombolysis with recombinant tissue plasminogen activator (rt-PA) within the first 4,5 hours after symptom onset [8]–[10]. However, recanalization efficacy is far from optimal, as only 20–40% of the patients treated with rt-PA achieve partial or full recanalization [11], [12]. On the other hand, rt-PA may provide additional benefits besides clot lysis. Indeed, it was recently shown that patients receiving rt-PA treatment had a better outcome even in cases of persistent MCA occlusion as compared to patients with persistent occlusion not receiving rt-PA [13]. This study aims to elucidate possible mechanisms of this finding in a new model of thromboembolic stroke closely mimicking human pathophysiology.

## Methods

This study was carried out in strict accordance with the recommendations in the Guide for the Care and Use of Laboratory Animals of the National Institutes of Health. All animal procedures were carried out strictly within German national laws and guidelines and were approved by the Ethical Committee for Laboratory Animal Experiments at the regional council in Karlsruhe, Germany. All surgery was performed under isoflurane anesthesia, and all efforts were made to minimize suffering.

### Thromboembolic Stroke

Thromboembolic stroke was induced by local injection of thrombin directly into the right MCA of male C57 black/6J mice (20–30 g) as originally described by Orset et al [14]. The mice were anaesthetized using 5% isoflurane and thereafter maintained with 1–2% isoflurane during the surgical procedure. An electric temperature probe was inserted into the rectum of the mouse to record the temperature, and found to be maintained at 37°C. A catheter was placed in the tail vein to allow the intravenous administration (200 µl) of vehicle or rt-PA.

The mice were placed in a stereotaxic device and the temporal muscle was retracted. A small craniotomy was performed, the dura was excised, and the middle cerebral artery (MCA) was exposed. The laser Doppler flow probe to measure cortical CBF was placed on the skull in the MCA territory. Finally, a micropipette filled with 1 µl of purified murine alpha-thrombin (1.5 UI) was introduced into the lumen of the MCA bifurcation and injected carefully to induce the formation of a clot *in situ.* The pipette was removed 10 minutes after the injection at which time the clot had stabilized.

CBF velocity was measured continuously by laser Doppler flowmetry allowing determination of spontaneous clot dissolution. A clot was defined as successful when the cerebral blood velocity decreased to a minimum of 60% from baseline at the time of thrombin injection and remained stable during at least 20 minutes. To induce thrombolysis, rt-PA (10 mg/kg;Actilyse) was intravenously administrated 20 or 40 minutes after thrombin injection. It was injected as 10% bolus and 90% perfusion during 40 minutes. The ability of rt-PA to induce thrombolysis was defined as effective when the cerebral blood flow returned to 60–100% of basal values. CBF was measured throughout the duration of the experiment (135 min). After 24 hours mice were euthanized and the brains were removed and frozen in isopentane.

### Magnetic Resonance Imaging

Imaging was performed on a 9.4T Biospec 94/20 USR (Bruker, Germany) small animal system equipped with 740 mT/m gradients and a 1H surface cryo probe (Bruker, Germany) [15] 24 h after MCAo. The animals were anesthetized with 1.5–2% isofluran and positioned into the magnet with a laser-controlled system for the animal cradles. Respiratory frequency and body temperature were monitored throughout the experiment and the latter was maintained with a water heating pad. The protocol consisted of a T2-weighted RARE sequence, a diffusion weighted multi-shot EPI sequence to obtain the apparent diffusion coefficient (ADC)-maps and a 3D flow-compensated gradient echo TOF angiography. Sequence parameters were set as follows. RARE: TR = 2.5 s; effective TE = 60 ms; echo train length = 4; 4 averages; matrix size = 384×384; FOV = 17×17 mm2; slice thickness = 0.4 mm; measurement time (12 slices) = 6 min 40 s. EPI-Diffusion: 4 phase encoding steps, TR = 3 s; TE = 20 ms; 4 averages; matrix size = 128×128; slice thickness = 0.4 mm; 3 orthogonal diffusion directions with three b-values b = 0, 100 s/mm2, 1000 s/mm2; measurement time (12 slices) = 5 min 36 s. 3D-TOF: TR = 22 ms; TE = 3.9 ms; Flip angle = 40°; matrix size: 256×256×128; FOV = 16×16×16 mm3; measurement time = 15 min 46 s. Angiograms were obtained by generating maximum intensity projections (MIPs) using ImageJ software (National Institutes of Health, Bethesda, MD). With the experimental setup and the described protocol it is possible to acquire T2- weighted images, ADC maps and TOF-MIPs within a measurement time of 28 min.

### Determination of Infarct Size

All animals were euthanized 24 hours after ischemia. Postmortem, 10 µm coronal cryosections were cut at 400 µm intervals and stained with the high contrast silver infarct method [16]. Focal edema in the ischemic hemisphere was determined according to Swanson et al [17]. Photographs of each coronal section were taken and analyzed using Image J. In addition, MRI was used exemplarily.

### Histological Examination

To evaluate the presence of hemorrhage after rt-PA treatment cresyl violet (Nissl staining) and diaminobenzidine (DAB) staining was performed on 10 µm coronal cryosections. In addition, hematoxylin-eosin-saffron staining was carried out for further neuropathological analysis. The neurons in the ischemic area were classified as intact or necrotic. The neurons were classified as necrotic when the cell showed swelling and demonstrated karyolysis, pyknosis, karyorhexis, cytoplasmic eosinophilia or loss of affinity for hematoxylin [18]–[20].

### Immunohistochemistry

For immunohistochemistry indirect immunofluorescence was used. The brains were removed and frozen in ice cold isopentane. They were then sectioned into 10 µm thick slices in a cryostat. The cerebral cryosections were fixed for 10 minutes in ice cold acetone and thereafter rehydrated in phosphate buffer solution (PBS) containing 0.25% Triton X-100 for 15 minutes. The tissue was then permeabilized and blocked for 1 hour in blocking solution containing PBS, 0.25% Triton X-100, 1% BSA and 5% normal donkey serum. The sections were incubated over night at 4°C with the following primary antibodies: rabbit polyclonal IL-6 (Abcam, ab6672), diluted 1∶200, rabbit polyclonal hsp 32 (Abcam, ab13243), diluted 1∶50, rabbit polyclonal hsp 70 (Abcam, ab2787), diluted 1∶50, rabbit polyclonal Caspase 3 (Abcam, ab4051), diluted 1∶50, rabbit polyclonal TNF-α (Abcam, ab6671), diluted 1∶200, rabbit polyclonal MMP9 (Abcam, ab7299), diluted 1∶400. All dilutions were done in PBS containing 0.2% Triton X- 100, BSA 1% and 2% normal goat serum. Sections were subsequently washed with PBS and incubated with secondary antibody for 1 hour at room temperature. The secondary antibody used was goat-antirabbit Alexa 488 conjugated (Invitrogen), diluted 1∶400 in PBS containing 0.2% TritonX-100 and BSA 1%. In addition, double staining with DAPI (KPL) 1∶5000 was performed. The sections were washed subsequently with PBS and mounted with mounting medium (Moviol). The same procedure was used for the negative controls but primary antibodies were omitted. The immunoreactivity of the antibodies were visualized and photographed with a Nikon confocal microscope A1R fitted with fluorescence optics at the appropriated wavelength.

Immunohistochemistry images were analysed using the ImageJ software (http://rsbweb.nih.gov/ij/). The fluorescence in different areas in each section was measured and a mean value was calculated. The ROI for the ipsilateral and contralateral sides was identical. These values are presented as percentage fluorescence in the ipsilateral compared to the contralateral group, where the contralateral side is set to 100%.

### Statistics

Data are expressed as mean ± standard error of the mean (s.e.m.), and n refers to the number of mice. Statistical analyses were performed with Kruskal-Wallis non-parametric test with Dunn’s post-hoc test, where P<0.05 was considered significant.

## Results

### Thromboembolic Stroke Model

**Figure 1:**
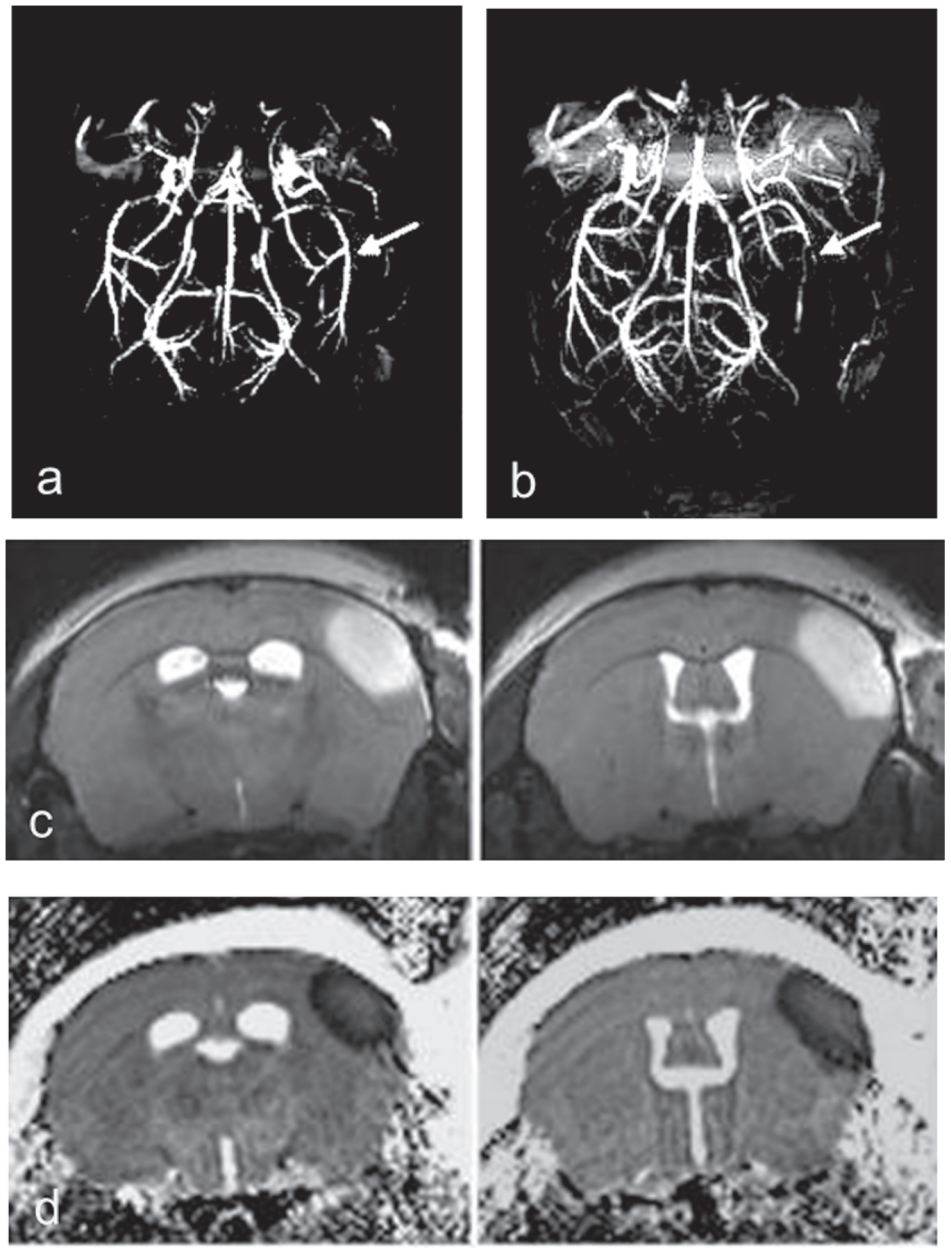
High resolution MR angiography of mouse brain before (a) and after (b) right MCA occlusion (arrows). T2-weighted images (c) and ADC-maps (d) demonstrating infarction.

Thrombin injection resulted in stable clot formation and cortical brain injury in 62% of the animals. MRI demonstrates the location of arterial occlusion and the resulting cortical infarction (Figure 1). In 38% of the animals we obtained a spontaneous recanalization within 10 minutes. The mortality rate was 32% and the animals died either during the surgery or overnight. There was no difference in the mortality rate between the groups.

### Effects of the rt-PA on CBF

**Figure 2:**
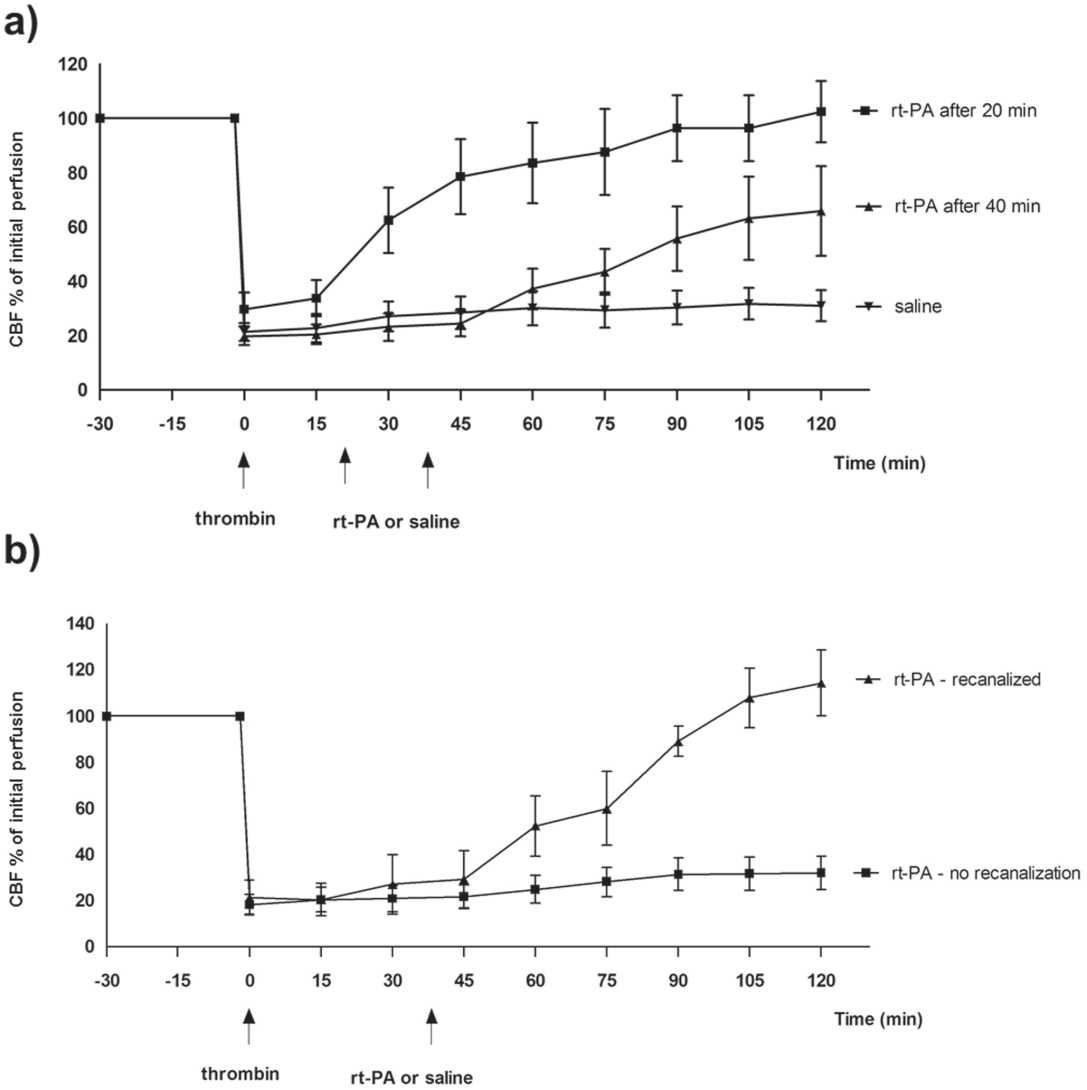
Effect of treatment with rt-PA on CBF after MCAO in mice. (a), MCAO+rt-PA after 20 min and 40 min, (b), MCAO+rt-PA after 40 min. Data were obtained by laser Doppler flowmetry and are expressed as mean ± s.e.m. values, n = 5–9 *P≤0.05, **P≤0.01, ***P<0.001 significant difference between treated groups and MCAO groups. Statistical analyses were performed using Kruskal-Wallis non-parametric test together with Dunn’s post-hoc test.

There is a significant variability in the response to rt-PA at different time-points after clot formation. At 20 min after clot formation, rt-PA treatment results in 100% recanalization (Figure 2a). However, rt-PA-induced thrombolysis only dissolves the clot in 38% of the animals (n = 8) when administered 40 min after clot formation (Figure 2a, b).

### Effect of rt-PA on Infarct Volume

**Figure 3:**
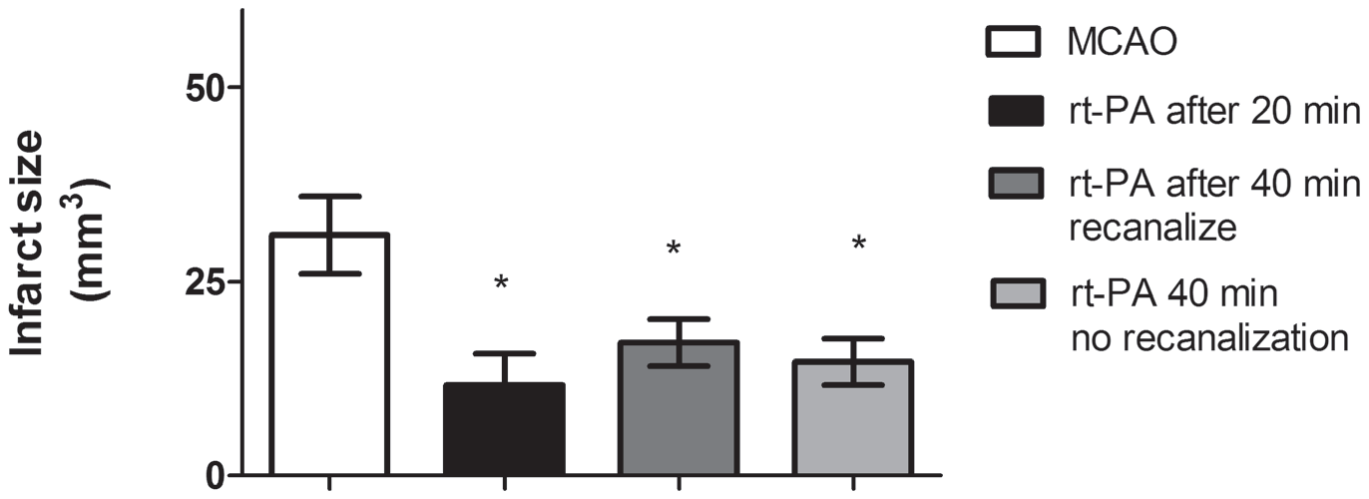
Size of infarct volume. Treatment with rt-PA significantly reduced the infarct volume compared to MCAO. Data are expressed as mean± s.e.m.; n = 5–9 *P≤0.05, significant difference between treated groups and MCAO groups. Statistical analyses were performed using Kruskal-Wallis non-parametric test together with Dunn’s post-hoc test.

Injection of thrombin resulted in an infarct that was restricted to the cortex with a mean lesion volume of 38,4±7,5 mm^3^. Treatment with rt-PA resulted in a significant reduction in infarct size in all groups, regardless of whether MCA thrombolysis was successful or not. (Figure 3). No edema was detected for any of the groups.

### Effect of rt-PA on Hemorrhage

Hemorrhages were examined histologically by Nissl and diaminobenzidine staining. In the MCAO group 78% of the animals showed no haemorrhage, while in the other 22% of the animals only very mild signs of hemorrhage were seen at 24 hours after stroke onset. Tissues of the group treated with rt-PA 20 minutes after clot formation showed similar results as the MCAO group, with mild signs of hemorrhage in 25% of the animals and no hemorrhage in 75% of the animals. Administration of rt-PA 40 minutes after clot formation resulted in mild signs of bleeding in 42% of the animals and no hemorrhage in 58% of the animals. The mild bleeding was similar in all groups examined, and mainly detected in the boundary of the infarct.

### Effect of rt-PA on Inflammation, Stress Factors, Metalloproteinase and Apoptosis

**Figure 4:**
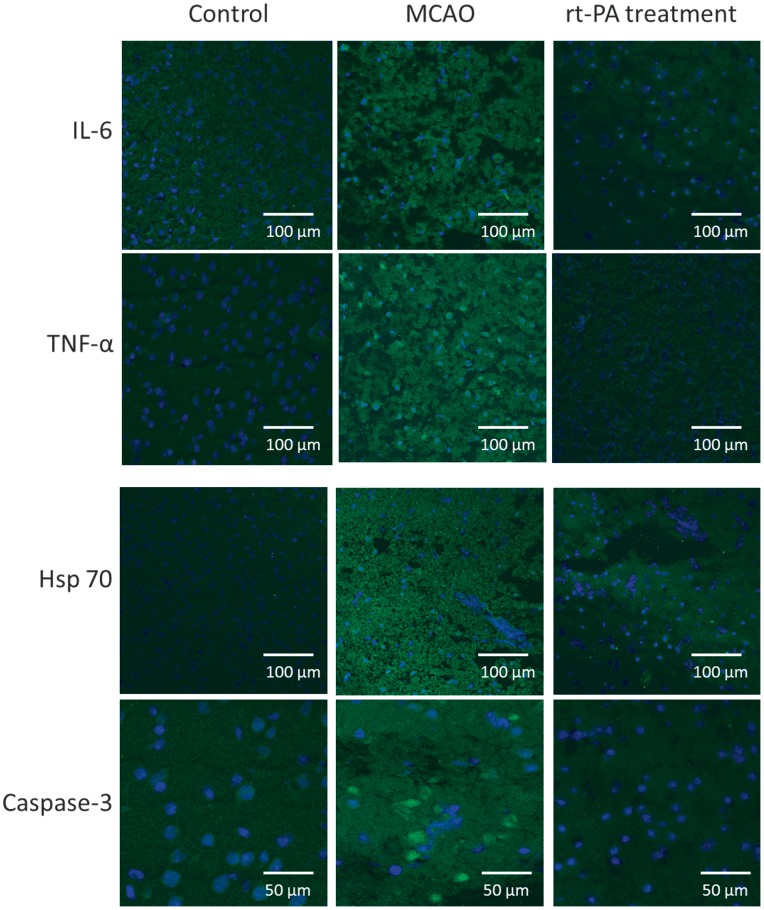
Effect of administration of rt-PA on protein expression. Sections from the infarction core showing IL-6, TNF-α, Caspase 3, MMP-9, and hsp 70 protein expressions. The images represent the control group (A, D, G, J, M and P), MCAO (B, E, H, K, N, Q) and MCAO+rt-PA treatment (C, F, I, L, O, R). There are significant increases in IL-6, TNF-α, Caspase 3 and hsp 70 protein levels in the MCAO group compared to the control group. Treatment with rt-PA prevented the increase in expression of these proteins in those animals that recanalized after treatment. There was a slight increase in hsp 32 and MMP-9 protein expression in ischemic brain tissue as compared to control. Data were obtained with confocal microscopy.

**Figure 5:**
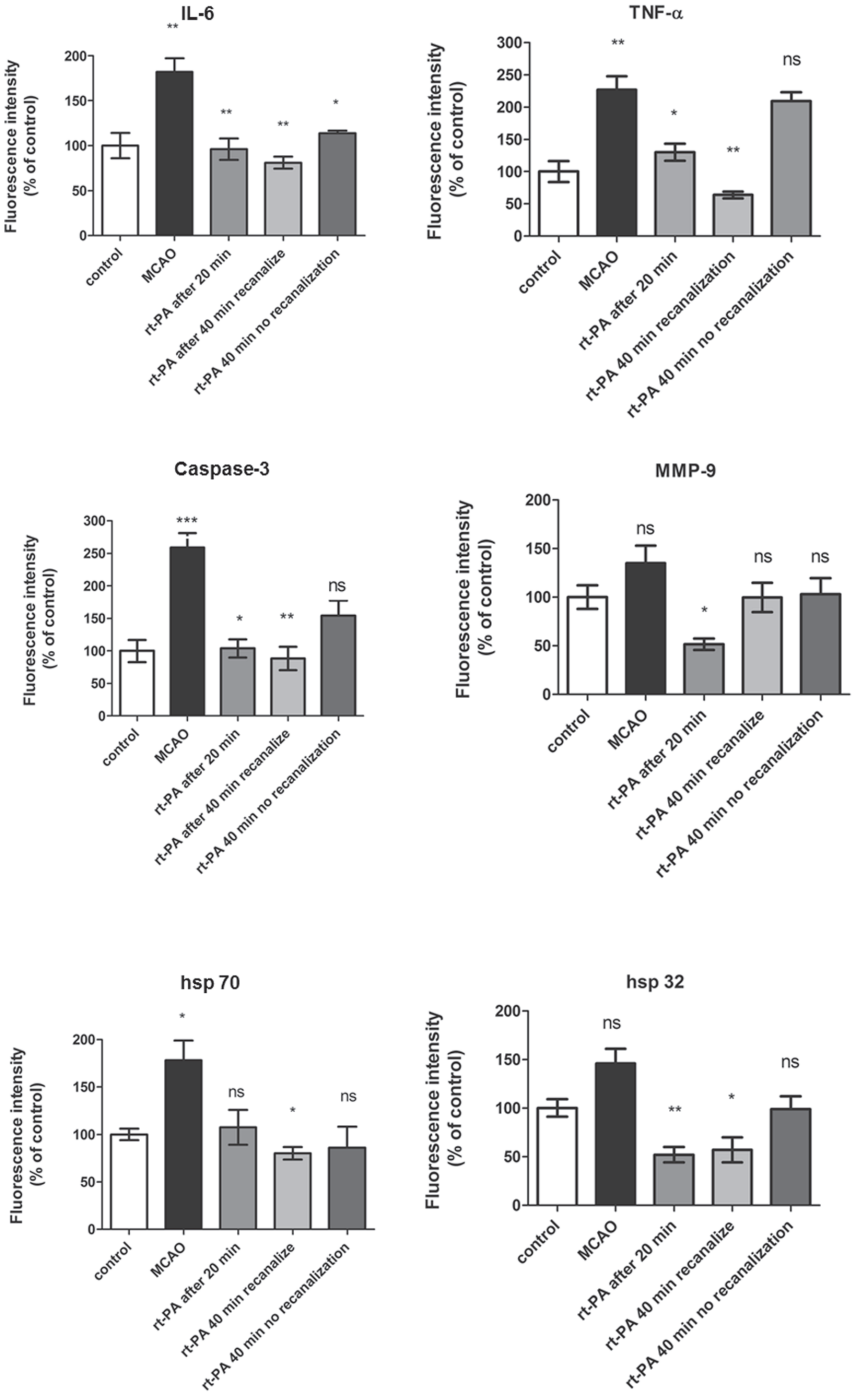
Bar graphs showing semi-quantification of fluorescence intensity for IL-6, TNF-α, Caspase 3, MMP-9, hsp 70 and hsp 32 proteins. Data are presented as the mean percentage relative to control ± s.e.m.; n 5 = 9 *P≤0.05, significant difference between control groups and MCAO, # P≤0.05, significant difference between treated groups and MCAO groups. Statistical analyses were performed using Kruskal-Wallis non-parametric test together with Dunn’s post-hoc test.

The localization and activation of the protein levels were examined by confocal microscopy and immunocytochemistry using selective antibodies towards inflammatory mediators (IL-6, TNF-α), apoptosis (Caspase 3) metalloproteinase 9 (MMP-9) and stress factors (hsp 70, hsp 32. Double immunohistochemistry staining versus nucleus, were performed to verify the localization. IL-6, TNF- α, Caspase-3 and hsp 70 protein expressions were significantly increased in the infarct area as compared to the contralateral side (Table 1, Figures 4 and 5). There was a tendency of increase of MMP-9 and hsp 32 protein expression in the MCAO, however it was not significant. Treatment with rt-PA starting with administration at 20 min after stroke reduced the stroke-induced activation of IL-6, TNF-α, Caspase 3, hsp 32, hsp 70 and MMP-9 significantly in the MCAO. Similar results were obtained with rt-PA treatment starting at 40 min after MCAO in those animals that recanalized after rt-PA treatment (Table 1, Figures 4 and 5). The inflammatory factors, apoptosis and stress genes that are activated after MCAO were not reduced by rt-PA treatment in those animals that did not recanalize after the treatment. (Table 1, Figures 4 and 5).

**Table 1:** 

Protein	Control	MCAO	rt-PA after 20 min	rt-PA after 40 min - recanalized	rt-PA after 40 min - no recanalization
**IL-6 (%) ± s.e.m**	100±14,0	182,2±15,2	95,9±15,0	80,97±6,6	113,9±2,9
**TNF-alpha (%) ± s.e.m**	100±16,3	227,3±33,5	130,5±12,8	63,8±5,4	209,5±13,9
**Caspase-3 (%) ± s.e.m**	100±16,9	259,6±27,2	104,4±14,8	88,48±17,9	154,1±23,3
**MMP-9 (%) ± s.e.m**	100±17,0	135,9±17,9	51,8±6,3	99,61±15,1	103,2±16,3
**hsp 32 (%) ± s.e.m**	100±13,9	146,2±15,5	52,2±19,4	57,25±23,4	98,9±13,4
**hsp 70 (%) ± s.e.m**	100±12,8	178,6±21,2	107,6±18,3	80,14±6,6	85,9±22,3

## Discussion

The present study demonstrates a clear correlation of the functional CBF data and protein expression of inflammatory mediators, apoptosis and stress genes after rt-PA treatment. MCAO significantly increase the activation of IL-6, TNF-α, hsp 70 and Caspase-3. However this activation of protein expression did not occur in those animals that recanalized with rt-PA treatment as opposed to animals that showed no recanalization after rt-PA treatment. Quite intriguing is our observation that inhibition of the inflammatory factors, stress genes and apoptosis correlates with CBF. Furthermore, the infarct sizes do not correlate with the CBF data. As in the human situation there is a significant decrease in infarct size in all animals treated with rt-PA regardless of whether thrombolysis was successful or not [13]. These data indicate that even if we cannot observe a recanalization after rt-PA treatment it still has an effect. We postulate that this effect is mediated through an improvement in microcirculatory flow, which appears to be below the threshold for detection by laser Doppler. This is in agreement with the data reported by Nedelmann et al, which demonstrates that rt-PA treatment both alone and in combination with contrast-enhanced ultrasound provides beneficial effects at the level of the microvasculature [21]. Previous experimental studies have demonstrated that “no reflow” of the microvascular compartment promotes infarct development after MCAO [22], [23]. Even low doses of rt-PA might be beneficial since it might affect the infarct size which in turn can affect the functional outcome. In an experimental study with the intraluminal transient MCAO model in rats it was demonstrated that low doses of rt-PA (2,5–7,5 mg/kg) reduced the infarct volume, diminished the activity of matrix metalloproteinase (MMP) and decreased blood-brain barrier disruption. However, the degree of which thrombolytic therapy may improve the functional outcome is uncertain in this model. In patients receiving rt-PA treatment a better outcome was shown even if the MCA remained occluded [13]. García-Yebenes et al [24] found that the infarct size is dependent on reperfusion (62% of animals) after treatment with rt-PA given 20 minutes after induction of MCAO. In our study we rather obtained 100% reperfusion when rt-PA was administered at the same time point. However, when giving rt-PA at 40 minutes after MCAO, we observed a reduction of infarct size independent of persistent occlusion or vessel reperfusion. Apart from different times of rt-PA administration, the different results in these studies may be related to the use of the thromboembolic model in different mouse strains with possible differences in the efficacy of rt-PA or variable tissue collateralization. Our recanalization data is in agreement with the clinical situation where only 20–40% of the patients receiving rt-PA treatment recanalize [25], [26].

Several experimental studies have documented deleterious effects of rt-PA, which may offset the beneficial effects of rt-PA upon the microcirculation that we have observed in this study. These include interactions of rt-PA with the low density lipoprotein receptor-related protein (LRP), the N-methyl–aspartate (NMDA) receptor, and annexin-II both in glial cells and in neurons resulting in parenchymal cell death [27]. Further evidence indicates that rt-PA mediates an increase in the permeability/leakage of the BBB [28]. Clinically, the most serious complications after rt-PA treatment are hemorrhage and blood-brain barrier breakdown [28]. Cerebral ischemia/reperfusion leads to release of pro-inflammatory mediators (TNF-α and IL-6) which in turns leads to production of MMP by resident and infiltrating cells, which altogether increase BBB permeability [29]. One marker for BBB permeability is MMP-9 and studies have shown that MMP-9 is responsible for rt-PA induced parenchymal hemorrhages after stroke. Our results indicate that there is a tendency of increased MMP-9 after MCAO, which is significantly reduced after rt-PA treatment given at 20 min after induction of MCAO. However, rt-PA treatment after 40 min decreased the protein levels of MMP-9, but not significantly as compared to MCAO. We did not observe any increase in BBB permeability after rt-PA treatment at 20 or 40 min after MCAO.

A limitation of our study is the lack of definitive information on the status of the microcirculation following treatment with rt-PA. Application of MR perfusion-weighted imaging and arterial spin labeling techniques could provide more insight into understanding the pathophysiology of perfusion changes during persistent MCA occlusion under rt-PA therapy. However, MRI may not have the spatial resolution needed to visualize and quantify changes in the microvasculature [30]. High-resolution 3D nano-CT imaging, which allows analysis of the vasculature in microscopic detail [31], may be useful for quantification of alterations in the cerebral microcirculation after rt-PA therapy [21]. The simultaneous implementation of such imaging procedures with laser Doppler monitoring of CBF was not technically feasible in our study, however.

In conclusion, this study shows that rt-PA treatment decreases ischemic lesion volume, which indicates that the success of thrombolysis therapy is not limited to the recanalization of the arterial main stem occlusion. In addition, there is a clear correlation between the protein expression of inflammatory mediators, apoptosis and stress genes with the recanalization data after rt-PA treatment.
